# The effect of dynamic versus static visualizations on acquisition of basketball game actions: a diurnal study

**DOI:** 10.1038/s41598-023-45278-x

**Published:** 2023-10-23

**Authors:** Ghazi Rekik, Ghada Jouira, Yosra Belkhir, Mohamed Jarraya, Cheng-Deng Kuo, Yung-Sheng Chen

**Affiliations:** 1 High Institute of Sport and Physical Education, University of Sfax, Sfax, Tunisia; 2 Research Laboratory: Education, Motricité, Sport et Santé, EM2S, LR19JS01, High Institute of Sport and Physical Education, University of Sfax, Sfax, Tunisia; 3 Tanyu Research Laboratory, Taipei, 112 Taiwan; 4 Department of Internal Medicine, Taipei Veterans General Hospital Hsinchu Branch, Hsinchu County, 310 Taiwan; 5 Department of Internal Medicine, Hsiao Chung-Cheng Healthcare Group, New Taipei City, 220 Taiwan; 6 Department of Exercise and Health Sciences, University of Taipei, No. 101, Sec. 2, Zhongcheng Rd., Shihlin Dist., Taipei City, 111 Taiwan; 7 Exercise and Health Promotion Association, New Taipei City, 241 Taiwan

**Keywords:** Circadian rhythms and sleep, Cognitive neuroscience, Computational neuroscience, Learning and memory

## Abstract

This study aimed to examine the effect of time of day (TOD) on the acquisition of basketball game actions from dynamic and static visualizations in physical education students (novice practitioners). Participants were quasi-randomly assigned to three treatments (static pictures, enriched static-pictures, or video). Morning and late-afternoon sessions were conducted, involving study phases and immediate-recall tests [game comprehension (GC) test and game performance (GP) test]. Oral temperature (OT) and mood states (MS) were also measured. Compared to the morning, the results revealed that afternoon resulted in higher OT, higher negative MS (e.g., anxiety and fatigue), and lower positive MS (i.e., vigor) in all experimental conditions. Moreover, the results showed that: (a) GC and GP decreased throughout the day (regardless of treatments), (b) GC and GP were better with enriched static-pictures (with arrows) than with static pictures, at both TOD, and (c) the video resulted in better GC and GP than the two static presentations, at both TOD. This study (a) highlights the morning's superiority in the acquisition of motor skills from dynamic and static visualizations, due to mood disturbances and lower arousal levels, and (b) encourages basketball teachers to use video modeling by experts, particularly in the morning, for explaining tactical skills.

## Introduction

Dynamic visualizations such as “video modeling by experts” are rapidly gaining popularity in various computer-based learning environments, and there has been much expectation that these visual mediums are proving especially effective for motor skill acquisition^1^. This is probably because (a) they are capable to convey information about how to perform a motor skill in a perfect way^2^, and (b) they are often seen as attractive to students, which can improve their motivation and facilitate knowledge construction^3^. According to Grèzes and Decety^4^, action observation is a form of motor simulation that activates the motor system in the absence of explicit motor performance. The positive effect of action observation seems to reflect automatic activation of motor codes matching to perceived motor behaviors^5^. Moreover, when observing peer or non-peer models performing an action, we, as humans, are able to imitate the movement kinematics of the models, which is coded trough biological motion^6^.

However, research within cognitive load theory argues that complex videos involving motor knowledge may impose high cognitive demands (i.e., extraneous cognitive load resources) on learners’ working memory (WM), due to their transient nature (*the transient information effect*^7,8^). The transient information effect occurs with instructional videos providing a non-stop flow of perceptual information that do not stay visible on the screen for long^9^. Dealing with such external visualizations required learners to retain information from earlier frames in WM to be able to connect it with information presented on later frames, thus succeeding in building a coherent internal representation of the content-based instruction^10^. These cognitive processes can overwhelm WM and thereby, negatively affect learning performance^7,11^. Otherwise, the amount of the learner prior knowledge in the domain is a crucial part of effective learning from dynamic visualizations^12^. It is well known that prior knowledge is stored in long-term memory (LTM) as cognitive schemas, through experience and deliberate practice^13^. The development of domain-specific knowledge can effectively reduce WM overload by assembling a large amount of information elements into a single unit. As a result, learners with higher levels of prior knowledge will be able to fluently process the transient information displayed via dynamic visualizations, by identifying the crucial aspects and ignore the unimportant ones^8,14^. Therefore, the transient nature of dynamic visualizations will negatively affect learning, particularly in learners with lower levels of prior knowledge^12^ (i.e., the case of our study sample). In attempting to reduce the higher extraneous cognitive load caused by the transient information effect, cognitive load theorists have suggested “*the use of a simultaneous series of static pictures*” instead of videos^12^. A series of photographs represent specific steps/events extracted from the transient information displayed in video clips (e.g.,^15,16^). Learning from such kind of static visualizations relies on the ability to infer and imagine the changes occurred in each picture in order to understand the whole system/procedure^17,18^. Compared to a continuous video, a successive display of static pictures allows learners (a) to benefit from enough time to detect and process significant information and effectively integrate it in LTM, and (b) to review and compare different parts of the display as frequently as desired^19^.

During the last two decades, much research effort has been devoted to testing the effectiveness of instructional videos against static graphics in the acquisition of various motor skills^3,15,19^. For example, model-based videos have been observed to be better than a series of static pictures to acquire hand manipulative tasks such as tying knots^17^ and building 3D Lego figures ^9^. In physical education domain, research also indicates that digital videos were more beneficial than a series of enriched static-pictures (i.e., with motion-indicating arrows) for the acquisition of motor behaviors requiring the whole body such basketball tactical actions^20^, and Judo gestures^3^. The superiority of videos over simultaneous presentation of static pictures when learning such kind of knowledge (i.e., motor skills) was referred to as the human movement effect^21^. This effect has often been linked to the existence of an effective Mirror Neuron System (MNS), which is automatically activated when an individual observes another person performing a motor skill^12,21,22^. Accordingly, this underlying architecture is responsible for the human ability to learn motor behavior through imitation. Following a functional magnetic resonance imaging (fMRI) study^23^, the activation of MNS was stronger (a) in subjects who had better motor experience in an activity, and (b) when the observed motor skills are part of the motor repertoire of the observer (e.g., if the subject has acquired the motor knowledge necessary to perform the specific motor actions/movements). Consequently, it seems important to underline that the brain processes of action observation are modulated by the expertise and motor repertoire of the observer.

Learning is the result of a stimulus that causes a change in the behavior of an organism. Based on the retention time, this cognitive function is typically classified into (a) short-term learning or acquisition assessed by immediate tests, and (b) long-term learning assessed after a notable delay of days and/or weeks^24^, or after performing a set of interfering tasks^23^, and even when using transfer tasks^26^. In this framework, studies on human memory indicated that performances on tasks involving immediate recall of information are time of day (TOD) dependent. In fact, a little number of these scientific works^27–29^ have observed that the diurnal variations of short-term recall performances (STRP) varies in parallel with the circadian rhythms of subjective alertness and body temperature, with superior performance observed in the evening compared to the morning. In contrast, a large number of experimental studies have shown a deterioration in STRP from the morning to the afternoon/evening^30–34^. Evidence of this circadian variation was obtained since 1885, when Ebbinghaus^35^ reported that the number of trials required for participants to immediately recall serial lists of nonsense syllables increased dramatically from late morning to early evening. These findings have been later confirmed when assessing immediate retention of digit sequences^36,37^, a sound-recorded story^38^ and news stories^39,40^, indicating that morning is the best period to retain information for a short time. According to Gates^30^, the morning is, usually, the best period for immediate retention of information, and the afternoon drop in STRP is due to a mental fatigue. Rather than being more “mentally fatigued” in the afternoon, the decrease of STRP over the day has been also attributed to an increase in basal arousal level over the day^39–41^. The diurnal variation of basal arousal level (usually operationalized in terms of body temperature) led the authors of numerous studies to predict that the morning superiority in the immediate recall of new information is related to low arousal circumstances^31,39,43–49^. This explanation has been reinforced by the study of Blake^36^ who showed that immediate recall of digit numbers decreased across the day as the subject’s level of arousal increased, suggesting a negative relationship between STRP and basal arousal level.

Although the effect of TOD on STRP was established through various experimental prototypes, no previous research (to the best of our knowledge) has explored how varied TOD could affect motor skill acquisition from dynamic and static visualizations. The present experiment attempted to fill this knowledge gap in a physical education (PE) setting. In particular, this study aimed to examine the impact of three different visual mediums: (video, static pictures, and static pictures enriched with motion-indicating arrows) and TOD (morning and late afternoon) in the acquisition of game actions. Basketball was chosen given its dynamic/complex nature and various motor skills involving different parts of the human body (e.g., moving, passing, and screening). It was hypothesized that the morning presentation of all instructional materials will lead to superior STRP (i.e., higher comprehension game scores and higher game performance scores) compared to the late afternoon hours (*Hypothesis 1*). It was also hypothesized that video will lead to superior STRP compared to the two types of static pictures, at both TOD (*Hypothesis 2*).

## Results

Tables 1 and 2 show the results for the mood states questionnaire and oral temperature (°C).

**Table 1:** Oral temperature and mood parameters’ ANOVA output.

Variables	TOD (main effect)			Condition (main effect)			TOD × condition (interaction)		
	F ratio	*p*-value	${n}_{p}^{2}$	F ratio	*p*-value	${n}_{p}^{2}$	F ratio	*p*-value	${n}_{p}^{2}$
Oral temperature (°C)	120.05	*p* < 0.001	0.68	0.43	*p* > 0.05	0.01	0.04	*p* > 0.05	0.001
Total mood disturbance (a.u)	207.81	*p* < 0.001	0.78	0.44	*p* > 0.05	0.02	0.07	*p* > 0.05	0.002
*Anxiety* (a.u)	190.04	*p* < 0.001	0.77	1.29	*p* > 0.05	0.04	0.1	*p* > 0.05	0.003
*Depression* (a.u)	18.9	*p* < 0.001	0.25	1.73	*p* > 0.05	0.06	0.5	*p* > 0.05	0.02
*Fatigue* (a.u)	158.17	*p* < 0.001	0.74	1.09	*p* > 0.05	0.04	0.3	*p* > 0.05	0.01
*Anger* (a.u)	204.74	*p* < 0.001	0.78	1.1	*p* > 0.05	0.04	0.55	*p* > 0.05	0.02
*Confusion* (a.u)	39.25	*p* < 0.001	0.41	4.76	*p* < 0.05	0.14	0.08	*p* > 0.05	0.003
*Vigor* (a.u)	62.28	*p* < 0.001	0.52	0.8	*p* > 0.05	0.03	0.07	*p* > 0.05	0.003
*Friendship* (a.u)	11.80	*p* < 0.05	0.17	0.79	*p* > 0.05	0.03	0.03	*p* > 0.05	0.001

TOD, Time of the day; a.u, arbitrary unit; °C, the degree Celsius.

**Table 2:** Mean (SD) for oral temperature (°C) and mood parameters recorded under the three experimental conditions, at the two TOD.

	Video						Enriched static pictures						Static pictures					
	MO	AF	*p*	d	Δ	95% IC	MO	AF	P	d	Δ	95% IC	MO	AF	*p*	d	Δ	95% IC
OT (°C)	35.78 (0.59)	36.9 (0.47)	*p* < 0.001	2.09	+ 1.13	− 1.69 to − 0.56	35.61 (0.72)	36.78 (0.75)	*p* < 0.001	1.59	+ 1.18	− 1.76 to − 0.63	35.62 (0.8)	36.82 (0.51)	*p* < 0.001	1.79	+ 1.19	− 1.74 to − 0.61
TMD (a.u)	30.75 (10.54)	55.15 (8.21)	*p* < 0.001	2.58	+ 24.40	− 33.06 to − 15.74	31.45 (8.08)	54.6 (9.11)	*p* < 0.001	2.69	+ 23.15	− 31,81 to − 14,49	29.85 (9.46)	52.90 (6)	*p* < 0.001	2.91	+ 23.05	− 31.71 to − 14.39
ANX (a.u)	13.05( 1.72)	16.90 ( 1.34)	*p* < 0.001	2.5	+ 3.85	− 5.33 to − 2.37	13.45 (1.66)	17.15 (1.01)	*p* < 0.001	2.69	+ 3.7	− 5.18 to − 2.22	12.70 (2.01)	16.70 (1.31)	*p* < 0.001	2.29	+ 4	− 5.48 to − 2.52
DEP (a.u)	11.45 (5.77)	16.40 (4.91)	*p* < 0.05	0.92	+ 4.95	− 9.71 to − 0.19	10 (4.05)	14 (5.1)	*p* > 0.05	0.87	+ 4	− 8.76 to0.76	10.65 (5.75)	13.4 (4.03)	*p* > 0.05	0.55	+ 2.75	− 7.51 to2.01
FAT (a.u)	5.85 (1.68)	9.8 (2.04)	*p* < 0.001	2.11	+ 3.95	− 5.73 to − 2.17	5.25 (1.95)	9.8 (2.04)	*p* < 0.001	2.28	+ 4.55	− 6.33 to − 2.77	6.25 (2.28)	10.4 (2.54)	*p* < 0.001	1.72	+ 4.15	− 5.93 to − 2.37
ANG (a.u)	12.05 (1.28)	16.95 (1.72)	*p* < 0.001	3.23	+ 4.9	− 6.69 to − 3.11	11.90 (1.22)	16.25 (2.43)	*p* < 0.001	2.26	+ 4.35	− 6.14 to − 2.56	12.05 (1.28)	17.25 (2.3)	*p* < 0.001	2.79	+ 5.2	− 6.99 to − 3.41
CON (a.u)	5.8 (1.63)	8.9 (2.55)	*p* < 0.01	1.45	+ 3.1	− 5.51 to − 0.69	6 (2.21)	8.75 (1.79)	*p* < 0.05	1.37	+ 2.75	− 5.16 to − 0.34	7.4 (2.69)	10.1 (2.93)	*p* < 0.05	0.96	+ 2.7	− 5.11 to − 0.29
VIG (a.u)	17.45 (3.51)	13.80 (3.74)	*p* < 0.01	1.01	− 3.65	1.03 to6.27	16.8 (2.34)	12.85 (1.59)	*p* < 0.001	1.97	− 3.95	1.33 to6.57	17.55 (2.44)	13.45 (2.65)	*p* < 0.01	1.61	− 4.1	1.48 to6.72
FRE (a.u)	17.15 (4.82)	13.35 (5.42)	*p* > 0.05	0.74	− 3.8	− 1.58 to 9.18	16.30 (5.4)	12.9 (5.65)	*p* > 0.05	0.62	− 3.4	− 1.98 to 8.78	17.7 (4.9)	14.45 (5.36)	*p* > 0.05	0.63	− 3.25	− 2.13 to 8.63

MO, morning; AF, afternoon; OT, oral temperature; TMD, total mood disturbance; ANX, anxiety; DEP, depression; FAT, fatigue; ANG, anger; CON, confusion; VIG, vigor; FRE, friendship; a.u, arbitrary unit; °C, the degree Celsius.

Regarding the results of the mood states questionnaire, ANOVA revealed a significant effect of TOD for anxiety, depression, fatigue, anger, confusion, friendship, and total mood disturbance, and a significant effect of treatment only for confusion. No significant interaction (TOD × treatment) on mood parameters was observed (see Table 1). In each of the individual subscales, the post-hoc analysis showed that students exposed to the three treatments reported lower values of vigor, and higher values of anxiety, fatigue, anger, depression (without significant difference for enriched static-pictures and static pictures), confusion, and total mood disturbance, in the afternoon compared to the morning (see Table 2). Further analyses on all mood parameters indicated no statistical differences between the three treatments, either in the morning or in the afternoon (all *p*-values > 0.05).

For the oral temperature, ANOVA revealed a significant effect of TOD, a non-significant of treatment, and non-significant (TOD × treatment) interaction (see Table 1). The post-hoc analysis showed that students exposed to the three treatments have higher oral temperature in the afternoon compared to the morning (Table 2). Furthermore, no statistical differences between the three treatments were found, either in the morning or in the afternoon (all *p*-values > 0.05).

**Figure 1:**
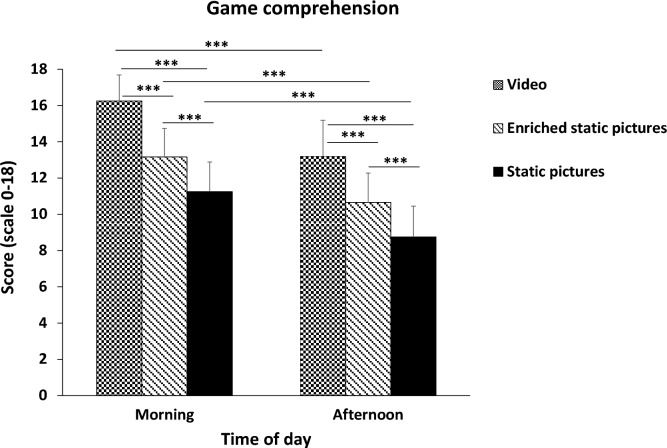
Game comprehension scores recorded in the morning and afternoon with the three treatments. *Note* *** present a significant difference at *p* < 0.001.

Concerning the game comprehension, ANOVA demonstrated a significant main effect of TOD (F _(1.57)_ = 70.57; *p* < 0.001,${n}_{p}^{2}$ = 0.55), a significant main effect of treatment (F _(2.57)_ = 80.01; *p* < 0.001,${n}_{p}^{2}$ = 0.74), and a non-significant (TOD × treatment) interaction (F _(2.57)_ = 0.33; *p* > 0.05,${n}_{p}^{2}$ = 0.01). As illustrated in Fig. 1, Post-hoc analyses showed that game comprehension scores were higher in the morning compared to the afternoon, with video (*p* < 0.001, ES = 1.76, Δ =  + 3.05, 95% CI = 1.35–4.75), enriched static-pictures (*p* < 0.001, ES = 1.56, Δ =  + 2.5, 95% CI = 0.80–4.20), and static pictures (*p* < 0.001, ES = 1.5, Δ =  + 2.5, 95% CI = 0.80–4.20). Further analyses indicated that game comprehension scores registered at the two TOD were higher with video compared to enriched static-pictures (in the morning; *p* < 0.001, ES = 2.04, Δ =  + 3.1, 95% CI = 1.44–4.76, and in the afternoon; *p* < 0.001, ES = 1.41, Δ =  + 2.55, 95% CI = 0.89–4.21), and compared to static pictures (in the morning; p < 0.001, ES = 3.24, Δ =  + 5, 95% CI = 3.34–6.66, and in the afternoon; p < 0.001, ES = 2.04, Δ =  + 4.45, 95% CI = 2.79–6.11). Additionally, the game comprehension scores were higher with enriched static-pictures compared to static pictures in the morning (*p* < 0.001, ES = 1.18, Δ =  + 1.9, 95% CI = 0.24–3.56), and afternoon (*p* < 0.001, ES = 1.14, Δ =  + 1.9, 95% CI = 0.24–3.56).

**Figure 2:**
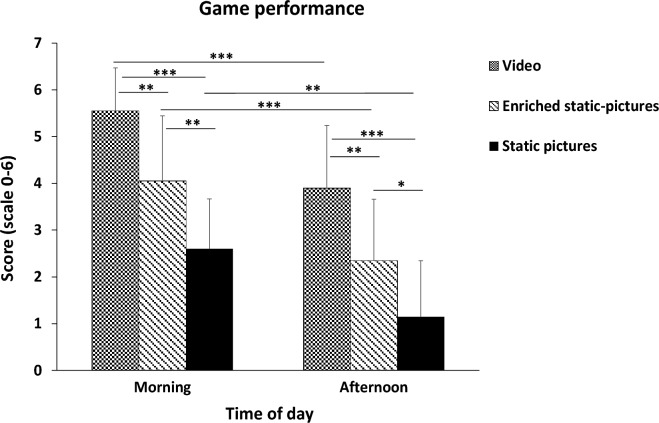
Game performance scores recorded in the morning and afternoon with the three treatments. *Note* *, **, and *** presents a significant difference at *p* < 0.05, *p* < 0.01, and *p* < 0.001 respectively.

About the game performance, ANOVA demonstrated a significant main effect of TOD (F (1.57) = 58.49; *p* < 0.001,${ n}_{p}^{2}$ = 0.51), a significant main effect of treatment (F (2.57) = 45.13; *p* < 0.001,${ n}_{p}^{2}$= 0.61), and a non-significant (TOD × treatment) interaction (F (2.57) = 0.13; *p* > 0.05,${ n}_{p}^{2}$= 0.005). As illustrated in Fig. 2, Post-hoc analyses showed that game performance scores were higher in the morning compared to the afternoon, with video (*p* < 0.001, ES = 1.44, Δ =  + 1.65, 95% CI = 0.54–2.76), enriched static-pictures (*p* < 0.001, ES = 1.25, Δ =  + 1.7, 95% CI = 0.59–2.81), and static pictures (*p* < 0.01, ES = 1.28, Δ =  + 1.45, 95% CI = 0.34–2.56). Further analyses indicated that game performance scores registered at the two TOD were higher with video compared to enriched static-pictures (in the morning; *p* < 0.01, ES = 1.27, Δ =  + 1.5, 95% CI = 0.29–2.71, and in the afternoon; *p* < 0.01, ES = 1.17, Δ =  + 1.55, 95% CI = 0.34–2.76), and compared to static pictures (in the morning; *p* < 0.001, ES = 2.96, Δ =  + 2.95, 95% CI = 1.74–4.16, and in the afternoon; *p* < 0.001, ES = 2.17, Δ =  + 2.75, 95% CI = 1.54–3.96). Additionally, the game performance scores were higher with enriched static-pictures compared to static pictures in the morning (*p* < 0.01, ES = 1.16, Δ =  + 1.45, 95% CI = 0.24–2.66), and afternoon (*p* < 0.05, ES = 0.96, Δ =  + 1.2, 95% CI =  − 0.01 to 2.41).

## Discussion

The current study aimed to explore the effect of TOD (morning vs. late afternoon) and instructional visualizations (video vs. static pictures vs. static pictures enriched with motion-indicating arrows) in the acquisition of motor skills in a PE context (i.e., basketball game actions). The study highlights the morning's superiority in the acquisition of motor skills from dynamic and static visualizations, due to mood disturbances and lower arousal levels. Moreover, the study confirmed the superiority of dynamic over static visualisations in learning motor skills at both TOD, due to the activation of the mirror-neuron system. To our best of knowledge, the present investigation is the first to reveal such pattern of results.

First, the results indicated that the morning presentation of all instructional materials led to better short-term learning compared to the late afternoon hours, thus confirming Hypothesis 1. In fact, learners performed better in the morning (between 08:00 and 09:00 h) rather than in the late afternoon (between 16:00 and 17:00 h), on the two immediate post-tests (i.e., game comprehension and game performance tests). These findings could be explained through the changes in mood states. Indeed, the present study results showed that learners indicated lower level of positive mood (i.e., vigor), and higher levels of negative moods (e.g., anxiety, fatigue, anger, and confusion), in the afternoon than in the morning. A mood disturbance is considered as a surrogate indication of mental fatigue^49,50^. Consequently, it is reasonable to predict that the morning superiority in the acquisition of basketball tactical skills resulted from an exacerbated mental fatigue after a busy school schedule (in the afternoon). Ebbinghaus^35^ and Gates^30^ supported this explanation, suggesting that the afternoon drop in STRP is due to a mental fatigue, and the morning is, usually, the best period to retain contents for a short time. Otherwise, the morning-afternoon effect on learning outcomes could be also explained following the arousal hypothesis, indicating that STRP mirrors rather than parallels the circadian arousal rhythm^32^. It is well known that basal arousal level increases across the day, to be superior in the afternoon/evening in comparison to the morning^40^. In this vein, the processing of new information becomes less efficient in the afternoon compared to the morning, as an optimal level of excitation is surpassed^44^. The arousal-related explanation was reinforced by Blake^35^ who observed that immediate retention of digit numbers decreased across the day as the subject’s level of arousal increased. In our study, oral temperature (physiological measure taken as indicant of arousal^48,52^) was lower in the morning than in the late afternoon. Thus, the morning superiority in the acquisition of basketball tactical skills (regardless of the visualization format) could be due the low arousal circumstances. The findings could fit with numerous earlier studies on human memory (out of computer-assisted motor learning environments), showing that performances on tasks involving immediate recall of information were better the morning rather than in the afternoon/evening. Indeed, the results patterns of the current study were previously found when evaluating immediate retention of serial lists of nonsense syllables^35^, digit sequences^36,37^, a sound-recorded story^38^, and news stories^39,40^.

Second, the results showed that video led to superior STRP compared to both types of series of static pictures, at the two TOD, thus confirming Hypothesis 2. Learners exposed to video demonstration achieved higher levels of game comprehension and game performance, in comparison with those exposed to enriched static-pictures and static pictures conditions, at both TOD. These findings are in line with Höffler and Leutner ’s meta‐analysis^52^, that is, dynamic presentation formats can be more effective than static formats if they are realistic and involve procedural-motor knowledge. The results are also consistent with a considerable amount of previous studies showing the superiority of videos over simultaneous presentation of static pictures in the acquisition of fine motor skills^9,15,16,53,54^. More importantly, the present study observations are in accordance with sport-related studies, indicating that dynamic visual supports are superior to their static counterparts in acquiring motor skills involving the whole body^3,55^. Indeed, the results found in this experiment could extend findings reported by Rekik and his colleagues^12,18,20^ to confirm the instructional benefits of videos (as opposed to still photographs) in the acquisition of basketball tactical actions, regardless of the TOD (morning or late afternoon). The findings are interesting in that they add some new insights about the special case for human movement instruction. Indeed, the possible influence of TOD in the acquisition of motor skills from videos and static pictures has not yet been explored. The present study results showed that the human movement effect^19^ remains valid in the morning and late afternoon, despite the decrease in learning over the day.

In spite of its speculative nature (as we did not report any neuro-physiological data in the results section), the automatic activation of the MNS seems a conceivable explanation for our results patterns. Indeed, the authors of this line of research studies have usually relied on the MNS hypothesis to argue the superiority of model-based videos over static pictures when learning about motor knowledge. According to Rajmohan and Mohandas^55^, the MNS is a group of specific neurons that “mirrors” the actions and behavior of others. In other words, the MNS is responsible for the human ability to acquire motor skills through imitation^22,56^. Moreover, as mentioned in the introduction, there is a strong activation of the MNS when the displayed motor skills are part of the motor repertoire of the observer^23^. As students are accustomed to performing the different actions of play (e.g., pass, shoot, and movements) during previous learning lessons of the basics of basketball, observing expert models who perform a coherent/complete playing system results in a strong activation of the brain processes of action observation and the cortical circuits that are involved in executing these actions of play. This assisted learners to deal with the transient nature of information and thereby, profit from the instructional video.

On the other hand, concerning the two presentations of simultaneous static pictures, it appears that the addition of numbered arrow-symbols in static pictures led to greater short-term learning outcomes. In fact, learners who received enriched static pictures were able to recall the game actions (either in a sheet of paper or on a basketball half-court) significantly better than those who received a series of static pictures, at the two TOD. Although it is important to understand what information they depict, arrows are considered one of the best mediums to describe dynamic changes^57^. Moreover, Garland and Sanchez^15^ suggested that the incorporation of arrows in multiple static pictures could facilitate the activation of MNS, which permit better learning performance. In team-sport domain, the provision of arrow symbols in static visualizations (e.g., diagrams) is crucial for conveying the essential game actions occurring on the pitch such as dribble, movement, and pass^25,58^. Consistent with the present study results, some earlier scientific works demonstrated the instructional benefits of using motion-indicating arrows with a series of static pictures. For example, it has been established that a series of static pictures enriched with motion-indicating arrows was more effective than a simple series of static pictures in conveying dynamic information about the structure of the cellular molecule and the processes of cellular respiration^59^. In physical education domain, H'mida et al.^3^ recently reported that enriched static pictures (with arrows) resulted in more efficient learning than the static pictures, with regard to acquisition and retention of the *Ippon-Seoi-Nage* (a Judo technique).

The current results could be consequent to the chosen protocol. First, the selected participants were mostly females, so that each experimental condition contained (n = 5) males and (n = 15) females. Previous studies have reported that females outperformed males with instructional videos, while both genders performed similarly with simultaneous static pictures, when learning about motor skills^60,61^. Thus, results could be different if experimental condition contained equal numbers of males and females. Second, only two test sessions (between 08:00 and 09:00 h *vs*. between 16:00 and 17:00 h) took place. It could be possible that additional testing sessions during the morning and afternoon hours might result in different outcomes. Third, only participants of “neither type” (according to his/her responses on the morningness-eveningness questionnaire) were included in the current study. Further investigations are required to replicate the study protocol among participants with different chronotypes, as circadian typology might affect memory performance^62^. Fourth, the study protocol was based on the conventional learning-and-recall situation^63^, in which participants were asked to perform learning tests (i.e. game comprehension test and game performance test) after they had just watched the computer-based materials. Therefore, findings could vary with other protocol (e.g., the ecological learning situation; see for example^14^. Lastly, core temperature of participants was measured (in this study) with a digital clinical thermometer inserted sublingually (i.e., oral temperature). It is important to point out that rectal and/or gastrointestinal temperature measurements are more reliable^64^. Consequently, more studies are needed on these specific aspects.

On theoretical levels, the current study (i) extends the research-base examination of the diurnal variation of STRP, indicating the morning superiority in the acquisition of motor skills from dynamic and static visualizations, due to mood disturbances and the lower arousal circumstances, and (ii) contributes to the growing body of knowledge on the human movement effect, while indicating its stability at different TOD due to the activation of the MNS that helps human to learn motor knowledge by imitation. Indeed, the present investigation mainly showed that (a) the acquisition of basketball game actions from dynamic and static visualizations decreases throughout the day, to be better in the morning (between 08:00 and 09:00 h) compared to the late afternoon (between 16:00 and 17:00 h), (b) the video led to better acquisition of basketball game actions when compared with the simultaneous static pictures (either enriched with arrows or without arrows), at the two TOD. Consequently, on a practical level, the findings encourage PE teachers to use instructional videos, particularly in the morning, for communicating tactical skills in basketball. Otherwise, additional educational research should perhaps explore the effect of video modeling by experts combined with motor imagery (e.g.,^65,66^) in the acquisition of other basketball game situations, and other team-sport playing systems (e.g., handball and rugby) to extend findings found here within the PE context.

## Methods

### Ethics approval

The present study was approved by the Committee of Protection of Persons of the University of Sfax (Sfax, Tunisia) with code number (C.P.P.SUD N° 0489/2023), and was conducted according to the international ethical standards for biological rhythm research on human beings^67^. All volunteers provided their written informed consent prior to participate in the study.

### Participants and design

**Figure 3:**
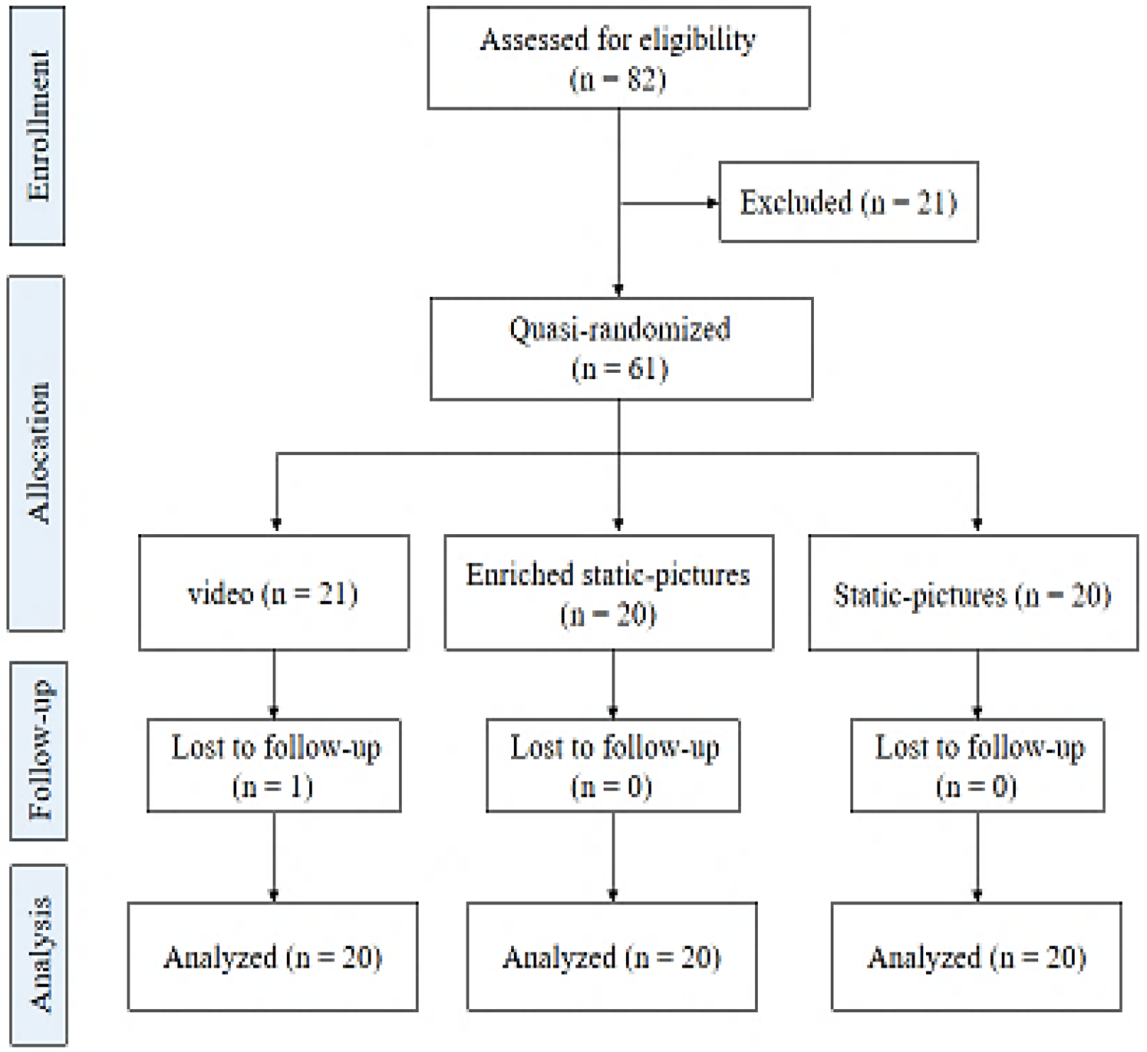
CONSORT flowchart of the study.

The sample size was a priori calculated using the G*power software (version 3.1; Düsseldorf, Germany^68^), and following the suggested procedure by Cohen^69^ for the F test (analysis of variation: repeated measures, within factors). Values for α err prob, power (1 − β err prob), and effect size f (V) were set at 0.05, 0.95, and 0.70, respectively. The G*power software indicated a minimal required sample size of thirty-nine participants. Yet, based on discussions between the research-team members (about possible participant attrition), we decided to exchange with at least sixty individuals about the involvement in the study. At first, eighty-two individuals were screened as potential participants for this study. Later, twenty-two were excluded, some failed to meet inclusion criteria, one did not provide an informed consent form, and one withdrew from the experiment (see Fig. 3). Eventually, sixty participants (mean age = 18.89 years, SD = 0.52; 75% females) voluntarily participated and completed the study protocol. Each of the participants was included in this study if:

- He/she is a first-year students from public universities in sports science,
- Aged between 18–20 years,
- Has never taken part in this type of experiment,
- Has normal or corrected to normal vision,
- Has never played or watching trainings or competitions in basketball,
- He/she is right-handed,
- He/she is classified as “neither type” according to his/her responses on the Morningness-Eveningness Questionnaire (MEQ^70^).
- He/she who does not reveal (a) a nap activity before afternoon sessions, (b) a lack of sleep the night before, or (c) a sleepless night,
- Has no shift work,
- Has no current muscular injuries,
- He/she is not addicted to drug, alcohol, nicotine or caffeine.

The study had a 3 (treatment: video vs. static pictures vs. enriched static-pictures) × 2 (TOD: morning vs. afternoon) factorial design, with repeated measures on the “TOD” factor to determine inter-condition and intra-condition differences. Participants were quasi-randomly (i.e., matched for gender) allocated to one of the three experimental conditions, so that each group contained equal numbers of males (n = 5) and females (n = 15).

### Apparatus and materials

#### Learning content

The learning content consisted of two offensive game systems in basketball (with similar levels of complexity). These scenes of play were designed in cooperation with two experienced basketball coaches/teachers (mean age = 43.1 years, SD = 0.84; mean experience = 11.25 years). Each game contained three players (a playmaker ①, a pivot ④, and a winger ③) who performed a coherent zone attack composed of nine different actions of play (pass, screening, lateral movements, layup, etc.), which were distributed across three steps. The playing system started when the team set up an attack, and ended when one of the players made a layup while the other teammates move for a rebound and/or a new game restart.

#### Computer-based materials

The apparatus consisted of an Acer aspire E15 Laptop (Taipei, Taiwan) placed at a distance of 30 cm from the viewing participant (with a 45° viewing angle). Three versions from the learning content were developed depending on whether corresponding temporal changes were provided explicitly (i.e., video) or not (i.e., static pictures and enriched static-pictures).

The video version was produced with a camera (using Samsung Galaxy Tab 3 SM-T211) placed above the ground from the middle of the field in an elevated position (approximately 2.5 m high). Each playing system was executed by three expert players (mean age = 21.7 years, SD = 1.26). The recorded videos were transferred (via a USB connector) and saved onto the computer. Additionally, they were resized to 820 × 972 pixels using Apowersoft Video Converter Studio software (version 4.8.4, Wangxu Technology, Hong Kong , https://www.apowersoft.com/video-converter-studio.html).

**Figure 4:**
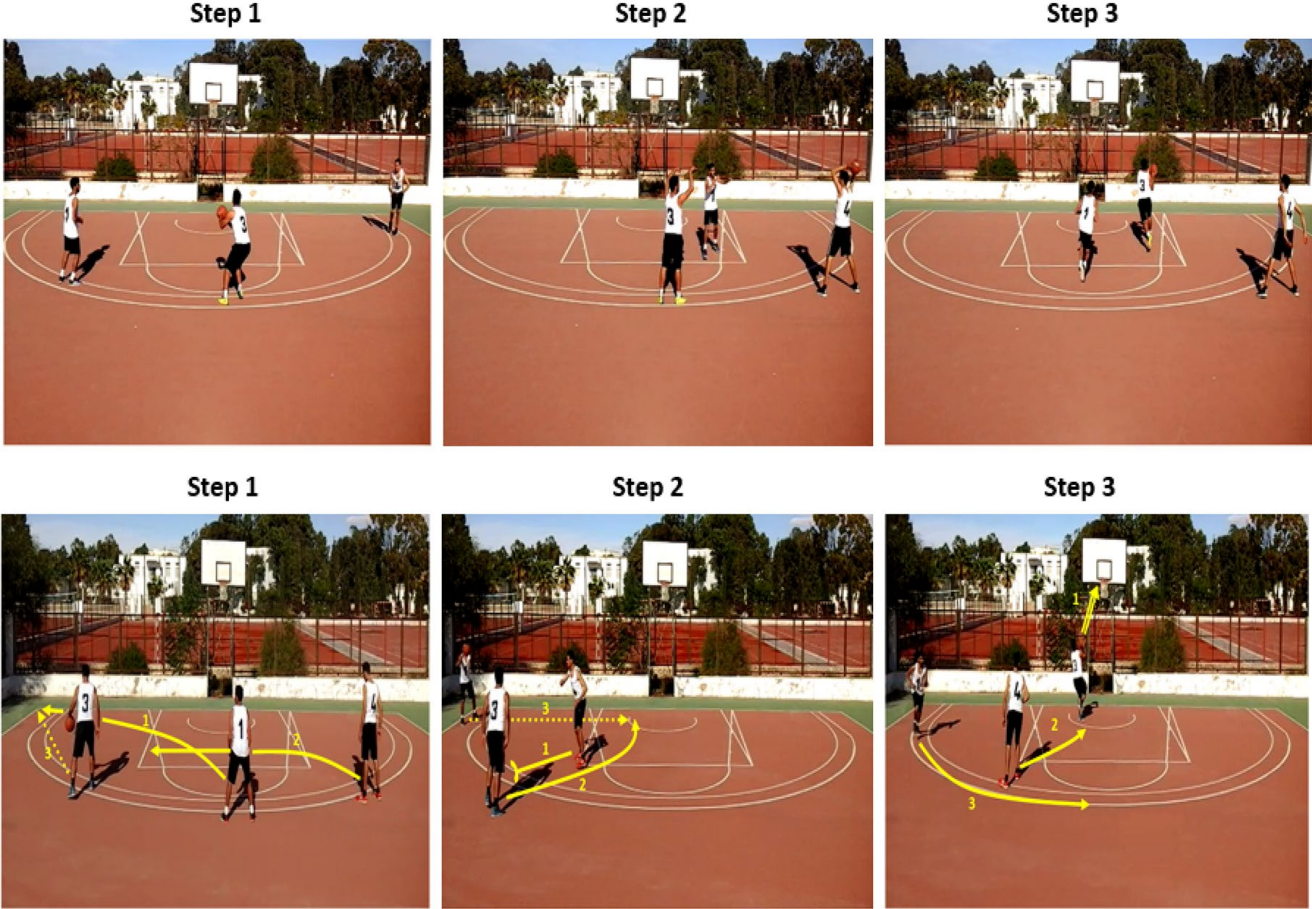
The simultaneous presentation of static pictures (top) and enriched static-pictures (bottom) showing the three key-steps of the two designed basketball-playing systems.

For both static-presentation versions, three key frames derived from the video were selected by the same experts who initially designed the game situations. The sizes of Photographs were also 820 × 972 pixels, and they were displayed simultaneously in one row. Photographs were captured using FastStone Capture 6.7 software (Barcelona, Spain). The only difference between the two static presentations was that actions of play were indicated by yellow numbered arrow-symbols (using Microsoft Paint) in the version of enriched-static-pictures (see Fig. 4).

Note that all the computer-based materials (i.e., video and the two static presentations) were delivered as Microsoft PowerPoint presentations and lasted 8 s before vanishing from the screen. Moreover, the three instructional conditions were system‐paced and purely visual to ovoid the potential benefits of interactivity^71^ as well as the possible occurrence of the modality, redundancy or temporal continuity effects associated with the simultaneous use of visual and verbal information^16^.

### Procedure and tasks

A familiarization session (with the experimenters, used material/devices, and tests) was carried out with all the participants, one week before the experiment. After that, the experiment was run in groups of five participants during sessions of approximately 60 min, while respecting the school’s daily schedules. In each group, students individually attended two sessions (with only one session per day) in a quasi-random order: one in the morning (between 08:00 and 09:00 h) and one in the late afternoon (between 16:00 and 17:00 h), under one of the three experimental conditions (static pictures, or enriched static-pictures, or video). Note that (a) the playing system presented in the morning was different (but with similar level of complexity) to the playing system presented in the late afternoon, (b) the order of the three experimental conditions was counterbalanced within subjects, and (c) any masking effects for the consumption of coffee, energy drinks, napping, or even strenuous mental/physical activity before testing were verified.

Fifteen minutes before starting each session, the participants came to the university laboratory (with stable mean ambient temperature and relative humidity; 21.1 ± 1.1 °C and 44.3 ± 7.6%, respectively). The principal investigator gives a brief description about the study protocol, objectives, and benefits. Afterward, the students moved, one by one, to an experimental box, equipped by an Acer aspire E15 Laptop (Taipei, Taiwan) placed at an empty desk with chair. At this moment, oral temperature was measured with a calibrated digital clinical thermometer (Omron^®^, Paris, France; accuracy ± 0.05 °C) inserted sublingually for at least 3 min in a seated resting position. Then, the student was given 5 min to complete the French version of the Profile of Mood States questionnaire (POMS-f^72^). After completing these two measurements, the student was invited to study twice the instructional material (depending on experimental condition), and he/she was instructed to memorize as precisely as possible the functioning of the playing system (study phase). Note that students exposed to the static pictures condition were initially informed of the functions of the arrows before watching the playing system. Immediately after the study phase, the participant was given 2 min to complete the game comprehension test. Finally, accompanied by a co-investigator, the participant individually moved to a basketball court (20 m away from the laboratory) to perform the game performance test for 1 min. The investigators controlled time using a hand-held stopwatch.

#### Oral temperature measurement

Oral temperature was measured with a digital clinical thermometer (Omron, Paris, France; accuracy + 0.05 °C) inserted sublingually for at least 3 min in a seated resting position.

#### Profile of mood states questionnaire

The POMS-f is composed of 65 words/adjectives and assessing seven emotional states. Participants responded to each word/adjective on a 5-point scale (“0 = not at all” to “4 = very strong”) with higher scores indicating a more negative mood. The Cranach’s alphas were 0.88 for the anxiety/tension, 0.91 for the depression/dejection, 0.84 for the anger/hostility, 0.76 for the confusion/aberration, 0.80 for the vigor/activity, 0.77 for the fatigue/inertia, and 0.81 for the friendship. The scores for all items, except interpersonal relationships, were taken into account to measure the total mood disturbance (TMD) following this calculation: TMD = (anxiety + depression + anger + fatigue + confusion) – vigor.

#### Game comprehension task

Participants were given a sheet of paper that included three schematic pictures of an empty basketball half-court representing the main-steps of the playing system. For each picture, the student was instructed to reproduce as accurately as possible the location of the players (by indicating their numbers) in relation to the ball already placed in its correct position. The student was also instructed to use the appropriate arrow symbols (from the caption below the schematic pictures) to indicate the actions of play. Each correct response yielded 1 point; otherwise, participants received 0 points. The scores could, therefore, range from 0 to 18. Two independent raters (i.e., basketball coaches/teachers who were blinded to the study design) assured the processes of coding, and disagreements between them were resolved by consensus.

#### Game performance task

Participants were instructed to reproduce as accurately as possible, on a basketball half-court, the actions performed by a randomly chosen player from the computer-based video (i.e., playmaker, pivot, or winger). The test was conducted with two professional basketball players (used as teammates) who already knew the functioning of the playing system. To guarantee the smooth running of the test, one of the basketball players was instructed provide verbal corrective feedback each time the student performed a wrong action. Student performances were recorded using a digital camera. Each correct position/action yielded 1 point; otherwise, participants received 0 points. The scores could, therefore, range from 0 to 6. Two independent raters (i.e., basketball coaches/teachers who were blinded to the study design) ensured the processes of coding, and disagreements between them were resolved by consensus.

### Statistical analyses

The Statistica software (version10; StataCorp LLC; college Station, TX, USA) was used for statistical analysis. The Shapiro–Wilk test of normality revealed that the data were normally distributed; therefore, parametric test was performed. A 3 (treatment: video vs. static pictures vs. enriched static-pictures) × 2 (TOD: morning vs. afternoon) ANOVA with repeated-measures on the last factor, was used for all dependent variables. When ANOVA revealed a significant difference, a post-hoc Bonferroni test was applied. The qualitative magnitudes were reported as partial eta squared ${(n}_{p}^{2})$ and Cohen’s mean standardized differences (ES) for post-hoc comparisons. The gain or decrease (i.e., afternoon values—morning values) for all parameters (Δ) and the 95% Confidence Interval (95% CI) were also provided for pairwise comparisons. An alpha level of 0.05 was used in reporting all statistical tests. Data are presented as mean ± standard deviation (SD).

## Data Availability

The datasets generated and/or analyzed during the current study are not publicly available due to confidentiality agreements, but are available from the corresponding author on reasonable request.
